# A Neglected Field of Study

**Published:** 1915-02

**Authors:** 


### EDITORIAL DEPARTMENT.

MRS. F. E. DANIEL, Publisher and Managing Editor.
DR. R. H. L. BIBB, Associate Editor.

CONTRIBUTING EDITORS:

Dr. H. Sheridan Baketel, New York, editor *Medical Times:* Dr. M. H. Boerner, Austin, Texas; Dr. G. Henri Bogart, Paris, Ill.; Dr. M. M. Carrick, Dallas, Texas; Dr. Edward H. Carey, Dean Medical Department, Baylor University, Dallas, Texas; Dr. W. L. Crosthwaite, Waco, Texas; Dr. T. D. Crothers, Hartford, Conn.; Dr. H. W. Cummings, Hearne, Texas; Dr. Oscar Dowling, New Orleans, President Louisiana State Board of Health; Havelock Ellis, M. D., London, Eng.; Dr. May Agnes Hopkins, Dallas, Texas; Dr. A. W. Fly, Galveston, Texas; Dr. G. B. Foscue, Waco, Texas; Dr. E. S. Goodhue, Holualoa, Kona, Hawaii; Dr. M. B. Grace, Seguin, Texas; Dr. Joe Gilbert, Austin, Texas; Dr. Charles H. Hughes, St. Louis, editor *Alienist and Neurologist*; Dr. James G. Kiernan, Chicago; Dr. George H. Lee, Galveston, Texas; Dr. John T. Moore, Houston, Texas; Dr. Frank Paschal, San Antonio, Texas; Dr. John Preston, Austin, Texas, Superintendent State Lunatic Asylum; Dr. G. Wilse Robinson, Kansas City, Mo.; Dr. Bacon Saunders, Fort Worth, Texas; Dr. Allen J. Smith, Philadelphia, Medical Department, University of Pennsylvania; Dr. Z. T. Scott, Austin, Texas; Dr. Ralph Steiner, Austin, Texas, President Texas State Board of Health; Dr. Walter S. Sutton, Kansas City, Mo.; Dr. John S. Turner, Dallas, Texas; Dr. Charles Venable, San Antonio, Texas.

---

### A Neglected Field of Study.

In the hospitals and dispensaries every effort of the physician is concentrated to understand the symptoms of the patient. Long ago the late Dr. Hammond of New York used to have his patients denuded and with hammer, pleximeter and stethoscope, thermometer and tape line, study every inch of the body and every possible reflex, mapping out on the surface whatever he found.

Of course the urine, the eye and the other senses were examined. The exhaustiveness of this examination, always impressed his patients, who were very willing to pay large sums for this study. It was a question afterwards what real facts could be obtained from such examinations, and how far they could be useful in determining the disease and the means for prevention.

In Chicago, years ago, a physician with a very large practice made minute inquiries into the hereditary history of the patient, his early life, culture, nutrition and vocation and what he was thinking about, and what success and failures he has had in his every-day life. He looked at the tongue, felt of the pulse, made little or no examination beyond that of pressing the patient to explain why he thought himself sick. This man had a tremendous practice, which paid him largely. Now which was the most scientific?

An eminent man, with an obscure disorder, went down to Johns Hopkins Hospital to be treated by experts of the highest skill and judgment in science. He was examined externally very minutely. The X-ray was turned on all the organs. Digestion with food tests were studied. The feces and urine were studied. The blood and serum from the spine were examined. The eyes, and all the senses were measured. From this data a diagnosis was determined.

The patient declared that no inquiries were made of his early life, hereditary history, and no studies were made of his mental work and how it had influenced his organism. His vocation, as a lawyer, and whether he had had any diseases, were about all the inquiries that had been made along that line. He was not asked his views, even on religion. The patient came away disappointed, and by advice went to the mountains of Tennessee. There he found a mountain physician who paid no attention to his external symptoms, but insisted on knowing what he was thinking about and what effect the various losses and successes of life had had on him.

The patient was startled at the minuteness in which his psychical life was examined and impressed that the physician could do him more good than any others had done. He followed his suggestions and became permanently restored.

The lesson from this is obvious. The sick patients who come to our care cannot be measured and weighed and understood by many of the so-called scientific processes of diagnosis.

Medical men are carrying the materialism of physical studies to an extreme and neglecting the mentality of the patient and the growth of abnormal thought, and its positive results on the organs. This is a neglected field.

The mountain physician was a far more exact scientific observer than the learned experts at Johns Hopkins. The Chicago physician had a clearer conception of disease than the late Dr. Hammond. The question occurs to every reader, are there not fields for study of disease and disease tendencies beyond the range of scientific measurements and so-called laboratory research? Practical physicians recognize this, and innumerable instances point out new fields which are yet to be occupied by the scientific man of the future.

T. D. C.

---

Good morning; have you thought what the Package Library might mean to you, Mr. Doctor Man? If you approve the plan and mean to take advantage of it, why don't you say so? It would take just about two minutes of your time to say what you think about it. I cannot know unless you write. Some of the JOURNAL'S progressive readers are enthusiastic about it and can hardly wait for me to get it in shape. Did you read the December issue? If you did not, then you do not know what the Package Library plan may eventually mean to the doctors of the State. Are you ever invited to contribute a paper to your medical society? Do you have difficulty in writing a paper because of the lack of information regarding the subject upon which you are preparing your article? The Package Library could be of inestimable value to you.

---

The following letter needs no explanation. We are glad that Mr. Lambert has spoken, for truly he speaks authoritatively. His reasoning is good and should appeal to every true American. Is it not high time that we were developing a national pride? Surely now is the psychological moment, and if we are ever to educate the public now is the time to begin. Let us begin with ourselves, and then may we speak to our friends and neighbors.—EDITOR.

### WHY WE SHOULD BUY GOODS MADE IN U. S. A.

DEAR MR. EDITOR: Collier's is doing commendable work in urging the American people to buy goods "Made in U. S. A." The future welfare of our country and the prosperity of our people depend very largely upon the success of the "Made in U. S. A." propaganda.

Many publications are encouraging the American people to buy American-made goods without giving specific reasons why they should do so. Don't you think that the movement will gain greater impetus and accomplish more for the manufacturer, as well as the consumer, if substantial "reason why" arguments are presented?

You are a student of national problems, while we manufacturers, who are engaged in import and export commerce, are deeply involved in the problems of manufacturing and marketing merchandise. I believe we should work together; therefore, I offer you some facts based on my personal experiences in European countries.

With my associates I am engaged in the manufacture of an article which is consumed in every part of the civilized globe. Our business originated thirty-three years ago and in its entirety is owned by American people. We maintain headquarters, offices and warerooms on this continent, also factories in many foreign countries, including France, Germany, Austria, and Spain.

Americans and Britishers always have traveled extensively.

Years ago they began to demand our product when abroad, and to satisfy this demand we attempted export shipments. Our American factories were then large enough to supply the universal demand, but when entering the foreign field we found it impossible profitably to clear our merchandise in many ports. Germany, Austria, France, and Spain demanded prohibitive duties. This left us the choice of discontinuing our export business or yielding to the demands of the foreigner, which in substance were: "If you want to do business in our country you must pay rents to our landlords, use our raw materials and employ our labor." We were practically forced to equip and maintain factories in the above mentioned places.

Without any desire to criticize the spirit which prompted the demands of these countries, I will say that the disadvantage to us has been very great. But we have had an even greater disadvantage to contend with, to illustrate which I will relate a part of my experience in Germany:

In Berlin I advertised for a highly trained city representative. Many responded and I interviewed and discharged all but two, who were requested to report to me the following Friday. When one man reported he immediately asked for his references, stating that he did not want the position. Upon being questioned he replied about as follows:

"I have spent the last two days interviewing the trade and investigating your product. It is American owned and the retailers will not push a foreign-owned product. Their customers prefer and demand articles made only in the fatherland. I see no future for me with your company."

The other man accepted the position but resigned shortly afterwards. He also found that German people demand goods made in the fatherland, *by concerns owned in the fatherland.*

Germany has asked us to buy her goods and we have cheerfully complied with the request. American dollars have made German manufacturers wealthy, yet the German people refuse to buy our goods.

Germany has become a strong nation because her people stick together and work together. They patronize home industry. Nowhere else in the world is the term "home industry" understood and appreciated as it is in Germany.

Many foreign-made cosmetics, proprietary medicines, textiles, toys and other articles are sold in America in competition with American-made and owned products that are really as good, and in some cases vastly better, at no higher price than the imported goods. Millions of dollars are expended by American people for French soaps, toilet articles, silks, millinery, gowns, etc. One great Paris firm does a tremendous toilet soap business in America, despite the fact that our domestic manufacturers produce superior soaps at less cost.

Within the last few years foreign manufacturers have established distributing points and even factories on American soil, *but they are foreign-owned.*

When we confine our demand for articles we eat, wear or use to those made in America by American capital and labor, then will our American enterprises grow in leaps and bounds, and since many foreign-owned articles are made in this country, or sold through domestic agents, each article should be carefully scrutinized and *its ownership determined*—so that those which are foreign-owned can be avoided whenever similar products of home manufacture are obtainable.

The German-American press and the agents of the kaiser are vigorously protesting against the lack of American sympathy for their cause, but perhaps it does not occur to them that our spirit of fair play, even our sense of humor, does not permit us to approve of or enjoy what may be styled a travesty on reciprocity—a burlesque on equity!

Foreign governments have done much to assure tariff protection of their industries, but in some countries it has rested with the people as individuals to do far more than it is possible to accomplish by stringent legislation. True patriotism means 100 per cent protection.

I am withholding the name of the company in which I am interested for the reason that I am not seeking free publicity for its product.

(The writer of the above letter is Mr. Jordan W. Lambert of the Lambert Pharmacal Company, St. Louis, manufacturers of Listerine. We give this information because the letter is fair, interesting and informative and we are glad to give the writer of it whatever benefit may accrue from our doing so.—E. C. P.)

We like the above letter because it gives real facts, based upon real experience. Americans are a tolerant, easy-going people in business matters, largely because prosperity generally has come to us without great effort. Our commerce of the future must be based upon more conscious effort, more skill in marketing and more patriotic support of our own industries, because more than ever we are going to face the effort, skill and commercial patriotism of foreign nations.

Foreign commerce is crippled now, but when the war is over it will meet us with redoubled effort in every market of the world, particularly our own. Therefore it behooves us to see that the goods we consume, wherever possible, are *made in the United States, and that they bear the name and trade-mark of the manufacturer as well as the national trade-mark, "Made in U. S. A."*

E. C. PATTERSON,
Vice President and General Manager P. F. Collier & Son, Inc.

---

Have you refused to write a paper for your society because you felt that your fund of information upon the given subject was too limited to permit of your writing an interesting paper? You need never do so again if the Package Library is established. Do you wish for its establishment?

